# Maneuvering Characteristics of Bilateral Amplitude–Asymmetric Flapping Motion Based on a Bat-Inspired Flexible Wing

**DOI:** 10.3390/biomimetics9030148

**Published:** 2024-02-29

**Authors:** Chuyi Lilong, Yongliang Yu

**Affiliations:** Laboratory for Biomechanics of Animal Locomotion, University of Chinese Academy of Sciences, Beijing 100049, China; lilongchuyi21@mails.ucas.ac.cn

**Keywords:** asymmetric flapping, MAV, aerodynamic, maneuver

## Abstract

Flapping-wing micro air vehicles (FWMAVs) have gained much attention from researchers due to their exceptional performance at low Reynolds numbers. However, the limited understanding of active aerodynamic modulation in flying creatures has hindered their maneuverability from reaching that of their biological counterparts. In this article, experimental investigations were conducted to examine the effect of the bilateral amplitude asymmetry of flexible flapping wings. A reduced bionic model featuring bat-like wings is built, and a dimensionless number $\Delta \Phi^{*}$ is introduced to scale the degree of bilateral amplitude asymmetry in flapping motion. The experimental results suggest that the bilateral amplitude–asymmetric flapping motion primarily induces maneuvering control forces of coupling roll moment and yaw moment. Also, roll moment and yaw moment have a good linear relationship. To achieve more efficient maneuvers based on this asymmetric motion, it is advisable to maintain $\Delta \Phi^{*}$ within the range of 0 to 0.4. The magnitude of passive pitching deformation during the downstroke is significantly greater than that during the upstroke. The phase of the peak of the passive pitching angle advances with the increase in flapping amplitude, while the valleys lag. And the proportion of pronation and supination in passive pitching motion cannot be adjusted by changing the flapping amplitude. These findings have important practical relevance for regulating turning maneuvers based on amplitude asymmetry and help to understand the active aerodynamic modulation mechanism through asymmetric wing kinematics.

## 1. Introduction

Since the concept of a micro air vehicle (MAV) was proposed, it has received much attention from organizations and research institutions all over the world because of its great potential for military and civil applications [1]. In general, MAVs can be classified into three types: fixed-wing MAVs, rotary-wing MAVs, and flapping-wing MAVs. With the miniaturization of the size, the deterioration in the lift-to-drag ratio for fixed-wing configurations will occur in the low Reynolds number regime [2]. As for rotary-wing MAVs, drawbacks such as low energy efficiency and high noise limit their ability to perform specific tasks [3]. Flapping-wing micro air vehicles (FWMAVs) inspired by flying creatures are highly maneuverable and efficient because of the unsteady forces associated with the flapping-wing flight [4]. These abilities are of great importance for obstacle avoidance and surveillance in confined areas or indoors.

Recently, many FWMAVs with better bionics and maneuverability have been developed [5,6,7,8,9,10]. Most of them are tailed FWMAVs, in which propulsion is achieved by flapping wings and direction is controlled by a tail. The relatively large tails compared to their sizes make them sensitive to wind gusts in low-speed flights [11]. Consequently, they cannot fly like their biological counterparts due to the lack of necessary agility, especially when flying forward at low speeds or when hovering. Practically, asymmetric wing kinematics (as a means to realize maneuvering flights) cannot be ignored. Without the use of thrust vectoring via a tail rudder, asymmetric control of wing kinematics can be utilized to generate asymmetric aerodynamic forces, and thus body torques.

Flight stability requires force and moment balances via bilateral symmetry in wingbeat kinematics [12], while flapping vertebrates may perform complex maneuvers through distinct wing kinematics. Cockatoos turning at low speeds displayed contralateral differences in wing kinematics [13]. Bats can vary wing kinematics to modulate aerodynamic forces [14]. For a dragonfly flight, leading-edge vortex formation enhancements and diminutions caused by asymmetric wing kinematics produce the torque necessary to complete maneuvers [15]. During the turn initiation phase of a hummingbird maneuver, large differences in stroke, pitch, and deviation angles are observed [16]. Flying creatures adopt different kinematic strategies to cope with different flight environments.

Among the many kinematic parameters, flapping amplitude and frequency are two important variables because animals vary the velocity of their wings by changing the frequency and amplitude of their wingbeat [17]. Meanwhile, an increase in amplitude is a predominant means of increasing the total aerodynamic force [12]. Therefore, the effect of bilateral amplitude asymmetry is worth discussing.

Bilateral amplitude asymmetry has been observed in animal flights. Henningsson et al. [18] found that through asymmetric amplitude, bats can obtain extra lift on the outward wing, causing a rolling motion in a turn maneuver. Windes et al. [19] reported that there is an amplitude difference of 10 to 20 degrees between contralateral wings during the turn. Inspired by these observations in animal flights, flapping mechanisms for MAV that are capable of achieving bilateral amplitude asymmetry have been developed. Finio et al. [4,20] presented a design that can asymmetrically change stroke amplitude with the control actuator displacement. Park et al. [21,22] introduced a flapping mechanism that can perform the in-phase amplitude–asymmetric flapping motion. Zhang et al. [23] proposed two flapping mechanisms with movable hinges providing adjustable flapping amplitudes.

However, it is challenging to guarantee controllability and repeatability in measurements performed on free-flying vertebrates due to individual variances and restrictions imposed by the animal’s flapping motion. Meanwhile, flying animals rarely change only one single variable at a time, which makes it difficult to distinguish the effect of individual kinematic variables [24]. As for the design of FWMAVs, the literature mainly focuses on the realization of asymmetric flapping motion, while systematic and quantitative research studies on the underlying mechanical mechanisms are still absent.

As a result, a bionic model with simplified bat-like wings is constructed in this article to investigate the effect of bilateral amplitude–asymmetric flapping motion. The design and construction of the flapping mechanism capable of executing bilateral amplitude–asymmetric flapping motion are described in detail. Methods of measuring and analyzing kinematics are presented. The transient and cycle-averaged aerodynamic forces are measured for different degrees of bilateral amplitude asymmetry and flapping frequencies. How contralateral wing movement asymmetries underlie aerodynamic force and torque generation is discussed. Passive pitching deformation of the flexible wing under different flapping amplitudes is also analyzed. The results provide meaningful control principles for FWMAVs.

## 2. Materials and Methods

### 2.1. Model Description

#### 2.1.1. Flapping Mechanism

**Figure 1:**
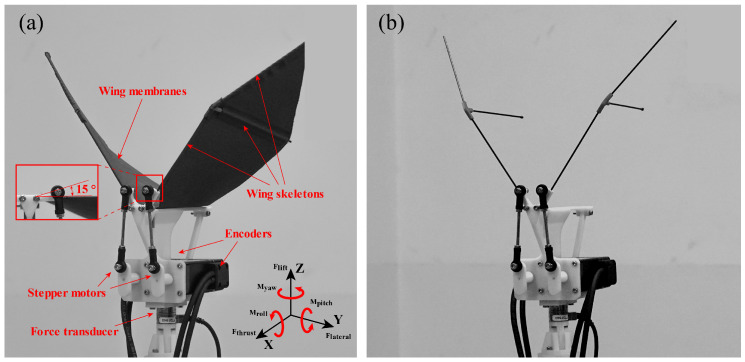
(**a**) Model with wing membrane, illustrating the earth-fixed coordinate definitions for the flapping model. The X-axis is parallel to the body’s longitudinal direction, indicating the front direction of the body; the Z-axis indicates the opposite direction of gravity; and the Y-axis along the lateral direction complies with the right-hand rule. (**b**) Model without the wing membrane.

An experimental model was constructed to determine the effect of bilateral amplitude–asymmetric flapping motion on aerodynamic forces. It comprises a body frame and a pair of flexible membrane wings actuated by two small stepper motors for each degree of freedom (one per wing, two total, see Figure 1a).

The simplified body frame and joints are printed from polylactic acid (PLA) on a fused deposition modeling (FDM) 3D printer. The printer has a resolution of 0.1 mm. The density and flexural modulus of the material are 1.3 $g/{\mathrm{cm}}^{3}$ and 1.9 GPa, respectively. The left and right wings are mirror-symmetric. Due to their good speed-torque characteristics, two two-phase closed-loop stepper motors with incremental photoelectric encoders were selected to drive the wings. The rotary encoder resolution is 1000 ppr (pulse per revolution). The two motors are set to rotate in counter-rotating directions to neutralize the moment caused by motor rotation. And the flapping frequency is controlled directly by altering the input step pulse frequency. The unsteady aerodynamic forces generated by the flapping wings were measured by a six-component force transducer (Nano17, ATI, Apex, NC, USA) with a range of 25 N and a resolution of 6.25 mN. The voltage signals from the transducer were transmitted through a data acquisition card (USB-6210, NI, Austin, TX, USA) and then converted into forces by using the calibration matrix.

#### 2.1.2. Wing Design

**Figure 2:**
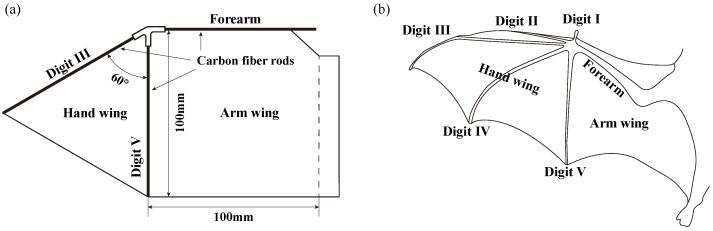
(**a**) Structure, layout, and dimensions of the simplified model wing; (**b**) schematic of the real bat wing, redrawn from the picture published by Hedenstrom and Johansson [26].

A real bat wing has up to 25 actively controlled joints and 34 degrees of freedom of motion [24], which means that it is difficult to model completely. In order to simplify the structure and reduce the complexity of the model design, the model used in this study only provides one degree of freedom of flapping motion. The degree of freedom of the pitching motion is passively realized by the aerodynamic and inertial forces of the flexible wing. Different parts of the bat wing can create different force generation [25]. Therefore, based on small- and medium-sized bats with high maneuverability, a bat-inspired wing was designed with hand wing and arm wing segments. The hand wing is an equilateral triangle with a side length of 100 mm, and the arm wing is a square with a side length of 100 mm, shown in Figure 2a. This wing planform design is geometrically simple while being bionic compared to the real bat wing (Figure 2b).

Wing membranes are made of ripstop nylon cloth with a 37 $g/m^{2}$ density and a 40 μm thickness. Wing skeletons are composed of carbon rods with a diameter of 1.5 mm. The trailing edge of the membrane is secured to the frame at the root, which refers to the characteristics of the wing-body integration of the bat. Bat wings are attached to the side of the body, from the neck to the ankle. This structure limits some of the freedoms of the bat wings, so bat wings cannot rotate freely like birds and insects when flapping. Morphological data for the bat-like wing planform can be seen in Table 1. The wing aspect ratio is defined as follows:

(1) $$AR=\frac{b}{\bar{c}},$$

where *b* is the wingspan and $\bar{c}$ is the mean chord. To calculate the wing mean chord $\bar{c}$, the wing area, *S*, is divided by the wingspan *b*:

(2) $$\bar{c}=\frac{S}{b}.$$

### 2.2. Kinematics Analysis

**Figure 3:**
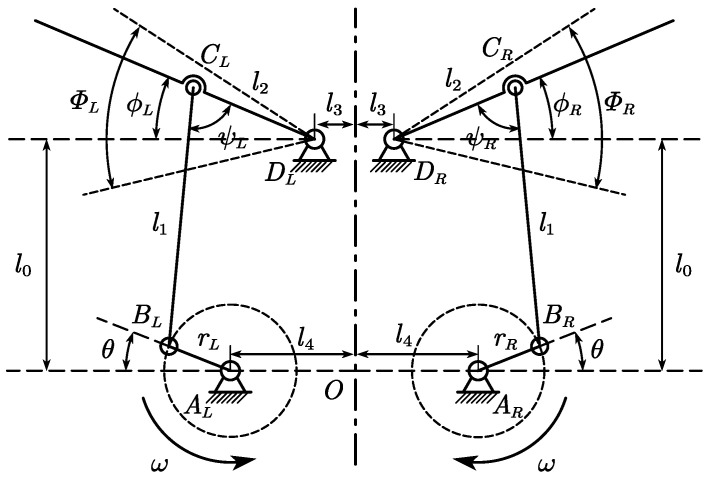
Schematic of the flapping mechanism, indicating definitions of the kinematics in the stroke (y-z) plane.

The transmission mechanism of the test device consists of two four-bar crank–rocker mechanisms that transform the rotational motion of stepper motors to a flapping motion (shown in Figure 3). The flapping mechanism has a vertical stroke plane.

The origin of the fixed coordinate system in the stroke plane is positioned at point O. Because the left and right are mirror-symmetrical, we use the right side as an example. The angle between ${\mathrm{OA}}_{R}$ and $A_{R}B_{R}$ is the rotation angle θ, positive counterclockwise. Similarly, the angle between the leading edge $D_{R}C_{R}$ and the horizontal line is the flapping angle of the right-wing $\phi_{R}$. The length of the crank $A_{R}B_{R}$ is noted as $r_{R}$, and the length of the connecting rod $B_{R}C_{R}$ is noted as $l_{1}$. More definitions of kinematic parameters are shown in Figure 3. The specific values of these parameters are shown in Table 2.

Note that there is a 15° angle deviation between the rocker $D_{R}C_{R}$ and wing leading edge in practice (shown in Figure 1a). Therefore, the data presented next are the data after considering this deviation. The kinematic analysis of the mechanism is performed to study the position of the flapping wings. The flapping phases of the left and right wings are set to be equal. According to transmission geometry, the relationship between the rotation angle, θ, and flapping angle, ϕ, can be derived. The flapping amplitude, Φ, and average flapping angle, $\bar{\phi }$, are written as follows:

(3) $$\Phi =\phi_{\max}-\phi_{\min},$$

(4) $$\bar{\phi }=\frac{\phi_{\max}+\phi_{\min}}{2},$$

where $\phi_{\max}$ and $\phi_{\min}$ are the maximum and minimum values of the flapping angle, respectively. Each wing (right or left) has its own $\phi_{\max}$ and $\phi_{\min}$. To scale the magnitude of bilateral amplitude asymmetry in flapping motion, the bilateral amplitude asymmetry coefficient $\Delta \Phi^{*}$ is introduced as follows:

(5) $$\Delta \Phi^{*}=\frac{\Phi_{\max}-\Phi_{\min}}{\Phi_{\max}},$$

where $\Phi_{\max}$ and $\Phi_{\min}$ are the largest and smallest flapping amplitudes for the right and left wings, respectively. They are defined as follows:

(6) $$\Phi_{\max}=\left\{\begin{array}{cc} \Phi_{\mathrm{RW}}, & \;\Phi_{\mathrm{RW}}\geq \Phi_{\mathrm{LW}}\\ \Phi_{\mathrm{LW}}, & \;\Phi_{\mathrm{LW}}\geq \Phi_{\mathrm{RW}} \end{array}\right.$$

(7) $$\Phi_{\min}=\left\{\begin{array}{cc} \Phi_{\mathrm{RW}}, & \;\Phi_{\mathrm{RW}}\leq \Phi_{\mathrm{LW}}\\ \Phi_{\mathrm{LW}}, & \;\Phi_{\mathrm{LW}}\leq \Phi_{\mathrm{RW}} \end{array}\right.$$

where $\Phi_{\mathrm{RW}}$ and $\Phi_{\mathrm{LW}}$ are the flapping amplitudes of the left wing and the right wing, respectively. By this definition, the value range of $\Delta \Phi^{*}$ is limited between 0 and 1. The larger $\Delta \Phi^{*}$ is, the greater the degree of amplitude asymmetry between the contralateral wings. The bilateral wings’ flapping amplitudes are equivalent and the two wings flap symmetrically when $\Delta \Phi^{*}=0$. When $\Delta \Phi^{*}=1$, it indicates that one wing is flapping while the other is not. The Reynolds number in this study is defined based on the mean chord length $\bar{c}$ and average wing tip velocity, ${\bar{U}}_{\mathrm{tip}}$:

(8) $$Re=\frac{\rho {\bar{U}}_{\mathrm{tip}}\bar{c}}{\mu },$$

(9) $${\bar{U}}_{\mathrm{tip}}=2\Phi_{\max}Rf,$$

where the air density is $\rho =1.225\;\mathrm{kg}/m^{3}$, the dynamic viscosity is $\mu =1.78\times 10^{-5}\;\mathrm{Pa}\cdot s$, *R* is the single wing length, and *f* is the flapping frequency.

**Figure 4:**
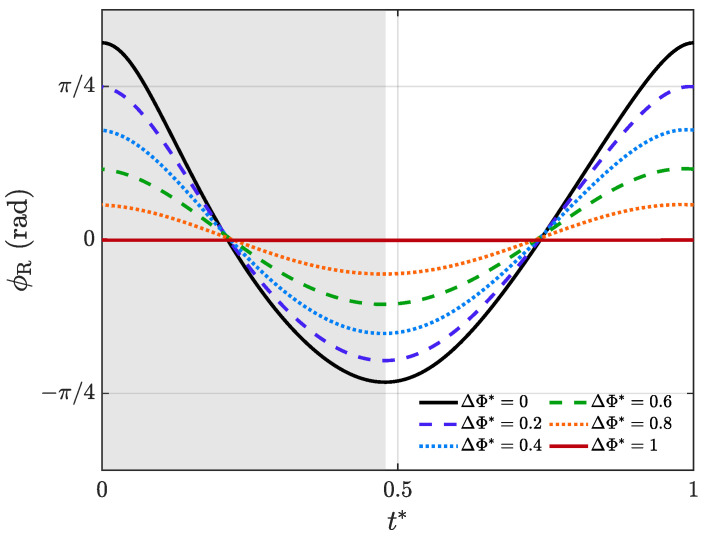
Rotation angle θ versus flapping angle, ϕ, under different asymmetry coefficients, $\Delta \Phi^{*}$. The gray background indicates the downstroke, while the white indicates the upstroke. And $t^{*}=0$ is the start time of downstroke.

**Figure 5:**
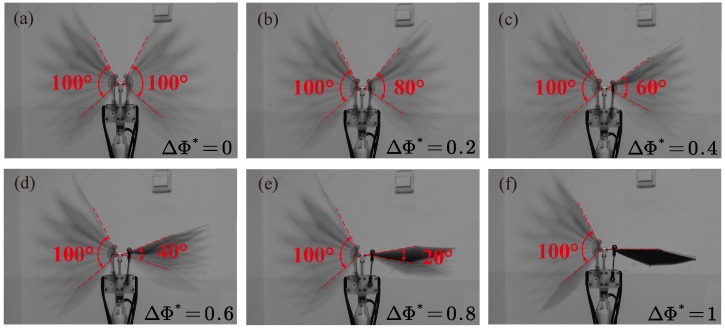
Snapshots of the flapping motion of the model with different $\Delta \Phi^{*}$. The left wing always flaps with an amplitude of 100°. The amplitude of the right wing ranges from 100° to 0° with a 20° interval. The photographs are extracted from the *f* = 3 Hz case during the upstroke.

Bullen and McKenzie [27] measured wingbeat frequency and amplitude for 23 species of bats, and found that maximum values for frequency were between 4 and 13 Hz, and maximum values for amplitude were between 90 and 150°. So, we choose 100° as our reference amplitude, which means that the left wing always flaps with an amplitude of 100°. To investigate the full range of the bilateral amplitude asymmetry coefficient, the amplitude of the right wing ranges from 0° to 100° with an increment of 20°; thus, $\Delta \Phi^{*}$ will vary from 1 to 0 with an interval of 0.2. Changing the length of the crank allows us to vary the amplitude. Detailed data can be seen in Table 3. Then the relationship between the flapping angle, ϕ, and non-dimensional time, $t^{*}=t/T$, under different $\Delta \Phi^{*}$ can be derived, as plotted in Figure 4. The normalized time begins at the initiation of the downstroke. *T* is the time period of one complete flapping cycle. The downstroke ratio $t_{\mathrm{down}}/T$ (where $t_{\mathrm{down}}$ is the time duration of the downstroke) is 0.48 for this flapping mechanism. This is consistent with the results of Busse et al. [28], who found that the downstroke ratio of living bats falls in the range of 0.40–0.52. Snapshots of the model performing the amplitude asymmetry flapping motion under different $\Delta \Phi^{*}$ are presented in Figure 5.

### 2.3. Experiment Setup

#### 2.3.1. Force Measurement

The model performs the asymmetric flapping motion in a forced hovering state at zero freestream velocity. The flapping frequency and bilateral amplitude asymmetry coefficient are two key kinematic variables in our experiments. Tests were performed over a range of asymmetric coefficients and flapping frequencies. And the tested frequencies range from 3 Hz to 5 Hz at 1 Hz intervals, which are common flapping frequencies of living bats [27]. Hence, the Reynolds number Re varies from 9960 to 16,600. The aerodynamic forces of asymmetric flapping motion were measured for different flapping frequencies *f* and amplitude asymmetry coefficients $\Delta \Phi^{*}$. The sampling duration for each case is 10 s. The sampling frequency of the force transducer is set at 4000 Hz with an average level of 4, resulting in an effective sampling frequency of 1000 Hz.

Lateral force, roll moment, and yaw moment, which are closely related to maneuvering flight, were extracted and analyzed among six components of forces and moments in each different case. In this study, lateral force is measured along the Y-axis, roll moment is measured along the X-axis, and the yaw moment is measured along the Z-axis. Forces and moments corresponding to the earth-fixed frame can be seen in Figure 1a. All the measured forces were transformed into the earth-fixed frame with the center of mass of the model as the origin. The relatively small oscillation of the center of mass caused by flapping motion is ignored. Both transient and cycle-averaged forces were analyzed and nondimensionalized as force coefficients. The transient lateral force coefficient, $C_{F-\mathrm{lateral}}$, roll moment coefficient, $C_{M-\mathrm{roll}}$, and yaw moment coefficient, $C_{M-\mathrm{yaw}}$ are written as follows:

(10) $$C_{F-\mathrm{lateral}}\left(t\right)=\frac{F_{\mathrm{lateral}}\left(t\right)}{\frac{1}{2}\rho {\bar{U}}_{\mathrm{tip}}^{2}S},$$

(11) $$C_{M-\mathrm{roll}}\left(t\right)=\frac{M_{\mathrm{roll}}\left(t\right)}{\frac{1}{2}\rho {\bar{U}}_{\mathrm{tip}}^{2}S\bar{c}},$$

(12) $$C_{M-\mathrm{yaw}}\left(t\right)=\frac{M_{\mathrm{yaw}}\left(t\right)}{\frac{1}{2}\rho {\bar{U}}_{\mathrm{tip}}^{2}S\bar{c}},$$

where $F_{\mathrm{lateral}}$, $M_{\mathrm{roll}}$, and $M_{\mathrm{yaw}}$ are the lateral force, roll moment, and yaw moment of the center of mass, respectively. Thus, the cycle-averaged lateral force coefficient, ${\bar{C}}_{\mathrm{lateral}}$, roll moment coefficient, ${\bar{C}}_{\mathrm{roll}}$, and yaw moment coefficient, ${\bar{C}}_{\mathrm{yaw}}$, are given by the following:

(13) $${\bar{C}}_{F-\mathrm{lateral}}=\frac{\int_{T}C_{F-\mathrm{lateral}}\;\left(t\right)dt}{T},$$

(14) $${\bar{C}}_{M-\mathrm{roll}}=\frac{\int_{T}C_{M-\mathrm{roll}}\left(t\right)dt}{T},$$

(15) $${\bar{C}}_{M-\mathrm{yaw}}=\frac{\int_{T}C_{M-\mathrm{yaw}}\left(t\right)dt}{T},$$

Measurements of 10 stable and successive flapping cycles were selected to evaluate the cycle-averaged forces and moments. To reduce the noises associated with the experimental environment and structural vibrations and prevent time-shifting of the data, a low-pass zero-lag fifth-order Butterworth filter with a cutoff frequency that was three times the flapping frequency was applied to the experimental results of the measured forces and moments [29].

#### 2.3.2. Inertial Subtraction

**Figure 6:**
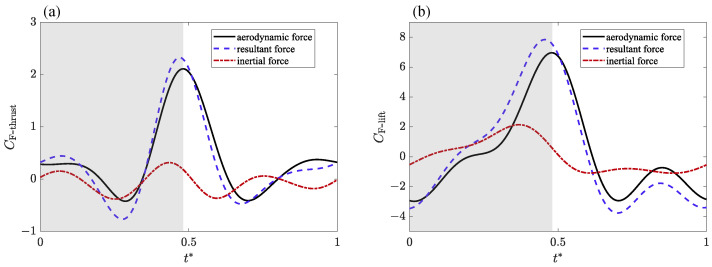
The resultant force, aerodynamic force, and inertial force variations in the case of flapping symmetrically, with an amplitude of 100° at a frequency of f=3 Hz during a flapping cycle. (**a**) Transient thrust force; (**b**) transient lift force.

When measuring the aerodynamic force generated by the model, it is necessary to consider the removal of the inertial force caused by the flapping motion [30]. The force transducer captures aerodynamic forces as well as inertial forces and mechanical vibrations. In order to remove the inertial force generated by the motion of the flexible wing from the measured resultant force, we used the flapping model without attached membranes (see Figure 1b) to repeatedly measure all test cases with other conditions unchanged, and the measured force included the sum of other inertial forces after ignoring the mass of wing membranes. The net transient aerodynamic force can be obtained by subtracting the force data without the wing membranes from the force data with the wing membranes. The resultant force, aerodynamic force, and inertial force variations in the case of flapping symmetrically, with an amplitude of 100° at a frequency of f=3 Hz, are shown in Figure 6.

#### 2.3.3. Kinematic Verification

**Figure 7:**
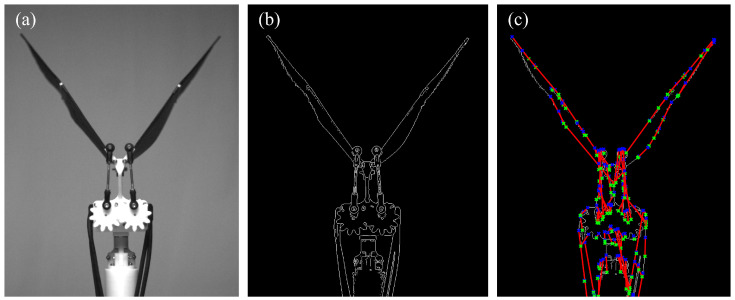
Process of the Hough transform. (**a**) Gray image; (**b**) edge detection; (**c**) detected line segments, the blue and green dots are the starting and ending points of the line segment, respectively.

In order to verify whether the flapping motion of the model is consistent with the set value, a high-speed camera (MIRO LC310, Phantom, Wayne, NJ, USA) is placed about 3 m in front of the model to capture the instantaneous position of the leading edge rod during the flapping process. The frame rate of the camera is 1000 fps, and the flapping angle, ϕ, is calculated by the Hough transform, which is a feature extraction technique that is often used to detect straight lines in digital image processing. The detection procedure is shown in Figure 7. Two gears are meshed to ensure that the two wings are flapping in phase. By opening holes at different radii on the gear disc, the crank length can be changed, which in turn changes the flapping amplitude. First, the edge detection is performed (Figure 7b) after the original image is grayed (Figure 7a), and then the Hough transform is performed on the image. The red is the detected line segment, and the blue and green dots are the starting and ending points of the line segment, respectively (Figure 7c).

**Figure 8:**
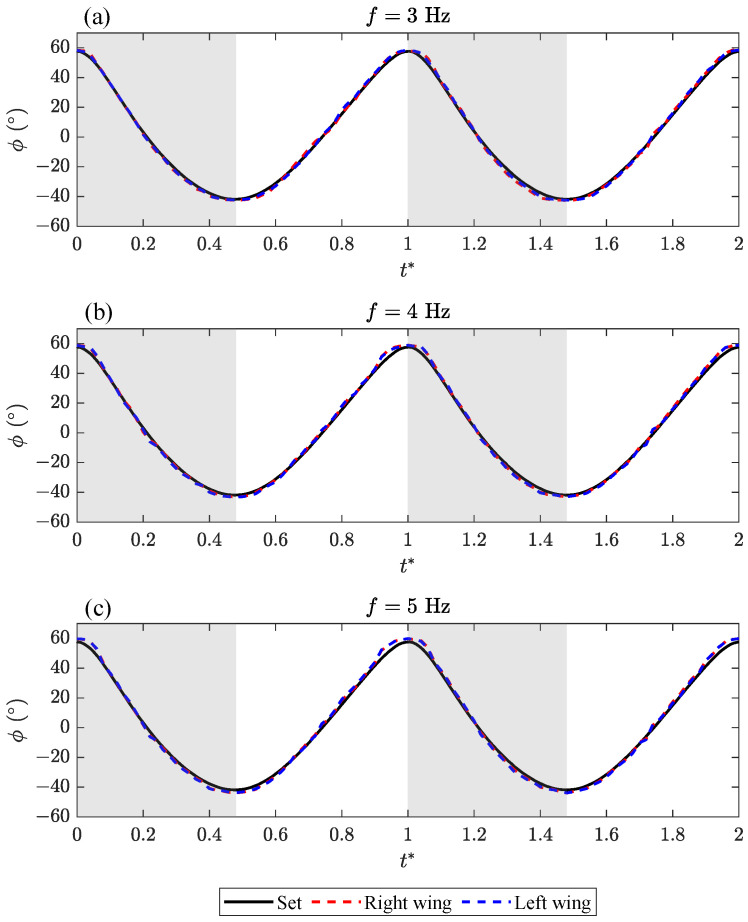
The variation in the flapping angle, ϕ, under three flapping frequencies (3, 4, 5 Hz) at $\Delta \Phi^{*}=0$.

The variations of ϕ under three flapping frequencies (3, 4, 5 Hz) at $\Delta \Phi^{*}=0$ were captured (see Figure 8). The results show that the flapping motions of the left and right wings are very symmetrical, and the phase angles are basically the same. In the case of a relatively small flapping frequency, the actual wing position is in line with the set conditions. The flapping amplitude, Φ, will increase slightly with the increase in flapping frequency, *f*.

## 3. Results and Discussion

### 3.1. Transient Aerodynamic Performance

The time-varying aerodynamic characteristics of bilateral amplitude–asymmetric flapping motion are analyzed and discussed. Transient lateral force, $C_{F-\mathrm{lateral}}$, roll moment, $C_{M-\mathrm{roll}}$, and yaw moment coefficients, $C_{M-\mathrm{yaw}}$ versus non-dimensional time, $t^{*}$, over a flapping cycle are presented for varying $\Delta \Phi^{*}$ under different *f*.

**Figure 9:**
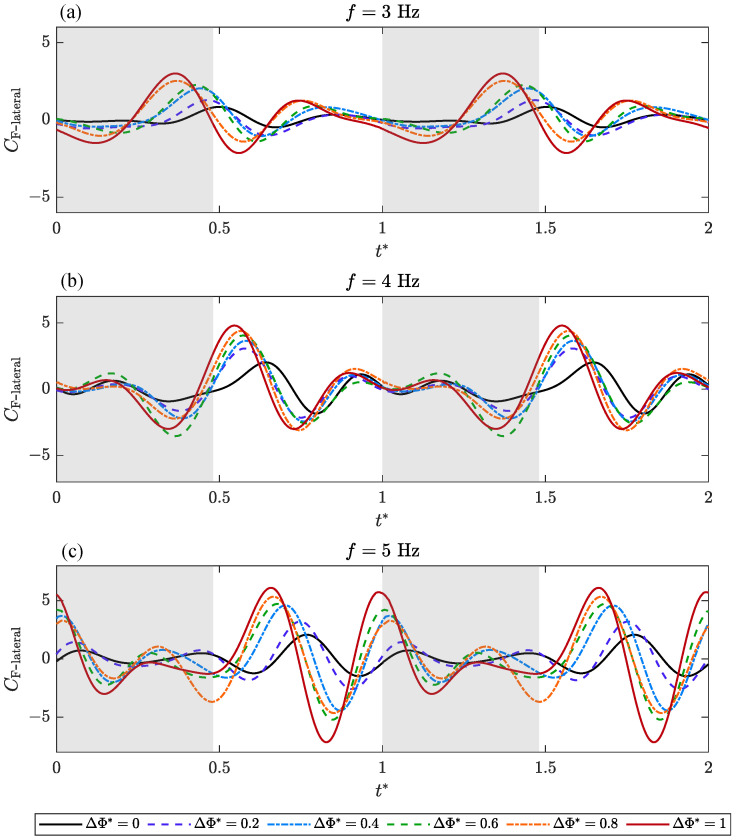
Transient aerodynamic coefficients of lateral force, depending on $\Delta \Phi^{*}$ and the flapping frequency, *f*, during a flapping cycle. The gray region indicates the downstroke.

In Figure 9, the variation in the transient lateral force coefficient, $C_{F-\mathrm{lateral}}$, with non-dimensional time, $t^{*}$, in two consecutive cycles, is plotted. In general, when the flapping frequency, *f*, is constant, the peak value of $C_{F-\mathrm{lateral}}$ increases with the increase in $\Delta \Phi^{*}$, and the time of the peak value gradually moves forward. When $\Delta \Phi^{*}$ is constant, the peak value of $C_{F-\mathrm{lateral}}$ increases with the increase in *f*, and the curve moves backward as a whole, which is opposite to the effect of the increase in $\Delta \Phi^{*}$. When *f* = 3 Hz (Figure 9a), the peak value of $C_{F-\mathrm{lateral}}$ is mainly generated near the lower reversal point ($t^{*}$ = 0.48), and a small positive peak appears near the middle of the upstroke ($t^{*}$ = 0.74). When *f* = 4 Hz (Figure 9b), the peak value of $C_{F-\mathrm{lateral}}$ moves backward to the vicinity of the initial stage of the upstroke. When *f* increases to 5 Hz (Figure 9c), the curve fluctuates more violently, and there is a large trough at the end of the upstroke, and a second peak is generated near the upper reversal point, which is almost equivalent to the first peak value.

**Figure 10:**
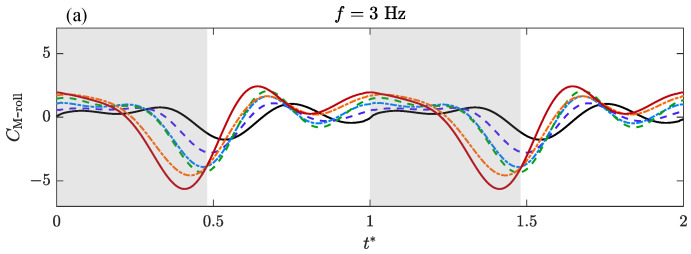

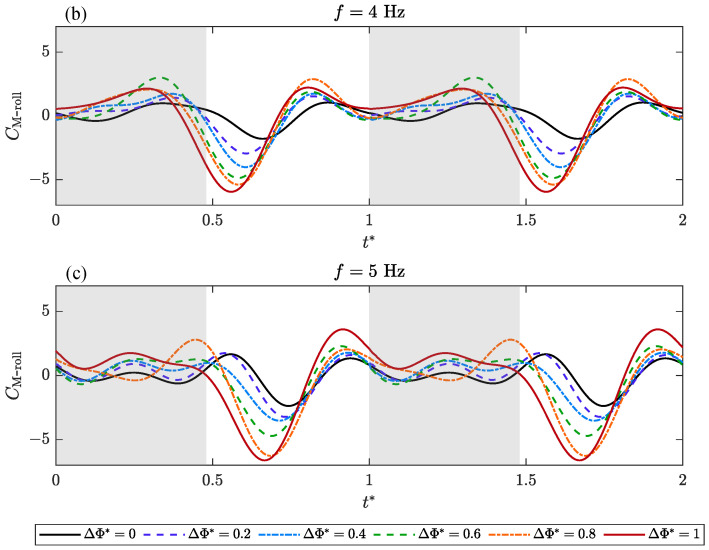
Transient aerodynamic coefficients of the roll moment depending on $\Delta \Phi^{*}$ and the flapping frequency, *f*, during a flapping cycle.

Figure 10 shows the variation in the transient roll moment coefficient $C_{M-\mathrm{roll}}$ with time. The roll moment can be regarded as the lift asymmetry of the two wings caused by flapping amplitude asymmetry. In the time history of a single cycle, the $C_{M-\mathrm{roll}}$ curve mainly produces a large trough. The influences of *f* and $\Delta \Phi^{*}$ on the $C_{M-\mathrm{roll}}$ are similar to that of $C_{F-\mathrm{lateral}}$. The increase in $\Delta \Phi^{*}$ makes the curve move forward and brings greater fluctuation amplitude. When *f* = 3 Hz (Figure 10a), the trough is generated near the lower reversal point. As *f* increases, the valley point moves backward to the initial section (Figure 10b) and the middle section of the upstroke (Figure 10c).

**Figure 11:**
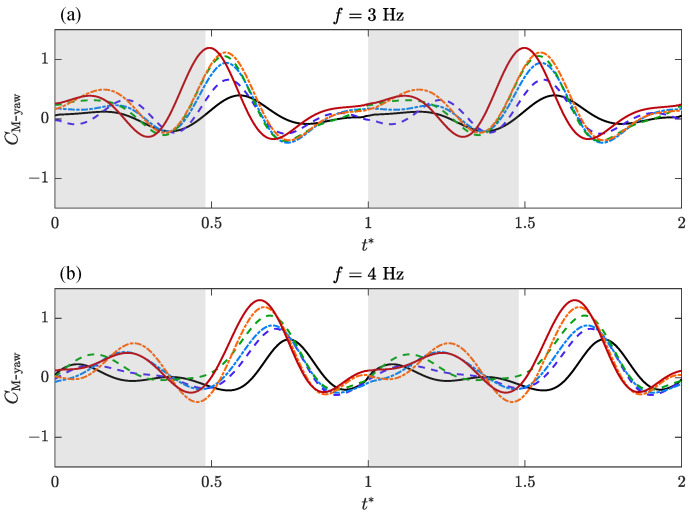

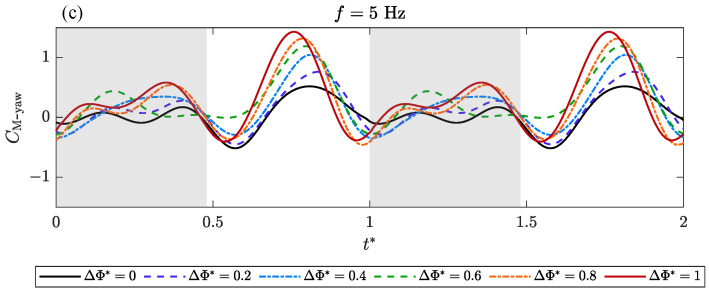
Transient aerodynamic coefficients of the yaw moment depending on $\Delta \Phi^{*}$ and the flapping frequency, *f*, during a flapping cycle.

The time-varying characteristics of yaw moment plots are shown in Figure 11. Similarly, the yaw moment can be seen as a result of the asymmetric thrust generated by the bilateral wings caused by flapping amplitude asymmetry. The transient variation in $C_{M-\mathrm{yaw}}$ is similar to that in $C_{M-\mathrm{roll}}$, but compared with $C_{M-\mathrm{roll}}$, the phase in $C_{M-\mathrm{yaw}}$ slightly lags behind and has a smaller fluctuation amplitude.

**Figure 12:**
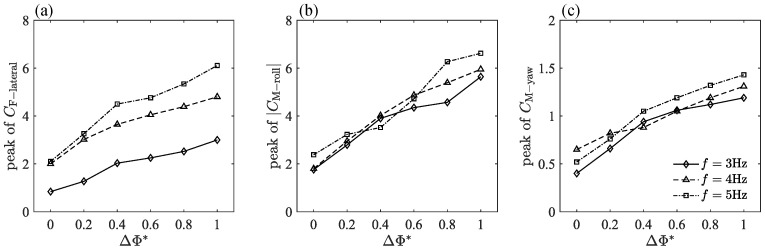
The variation in the peak value of the transient force with $\Delta \Phi^{*}$ at different *f*.

Furthermore, the variation in the peak value of the transient force with $\Delta \Phi^{*}$ at different *f* is plotted (Figure 12). The three force components have roughly the same change rule. The peak value increases with the increase in $\Delta \Phi^{*}$, but the increase in *f* has a more obvious gain effect on the peak value of $C_{F-\mathrm{lateral}}$ compared to $C_{M-\mathrm{roll}}$ and $C_{M-\mathrm{yaw}}$. The increase in *f* makes the peak curve in $C_{M-\mathrm{yaw}}$ move up slightly, and the peak in $C_{M-\mathrm{roll}}$ seems to be less affected by the change of frequency.

Based on the above analysis, the characteristics of transient aerodynamic forces are strongly dependent on $\Delta \Phi^{*}$ and *f*. When the frequency, *f*, is constant, with the increase in $\Delta \Phi^{*}$, that is, the increase in the amplitude asymmetry of bilateral wings, the peak value of the transient force gradually increases, and the extreme point gradually moves forward. When $\Delta \Phi^{*}$ is constant, the maximum transient force value also increases with the increase in flapping frequency, *f*. In the measured frequency range, the change in transient force caused by frequency is less than that caused by $\Delta \Phi^{*}$. Another difference is that as the frequency increases, the peak point of the transient force will move backward, which is opposite to the change caused by the increase in $\Delta \Phi^{*}$.

### 3.2. Cycle-Averaged Aerodynamic Performance

The net forces produced throughout the flapping cycle determine whether natural flyers can complete turn maneuvers agilely. Therefore, a cycle-average analysis of the mean lateral force, roll moment, and yaw moment was conducted to evaluate the overall aerodynamic performance of the bilateral asymmetric flapping motion and fully comprehend the global effects of $\Delta \Phi^{*}$ with varied *f*.

**Figure 13:**
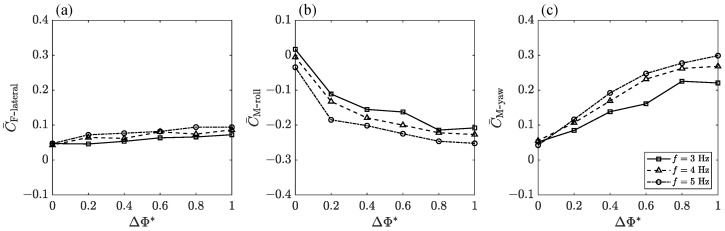
The effects of $\Delta \Phi^{*}$ and *f* on cycle-averaged aerodynamic performance.

Principally, the averaged lateral force ${\bar{C}}_{F-\mathrm{ateral}}$ increases slowly with the increase in $\Delta \Phi^{*}$, as shown in Figure 13a. The increase in frequency, *f*, will contribute to the overall increase in ${\bar{C}}_{F-\mathrm{lateral}}$, but this gain is not obvious in the measured frequency range. Thus, it may not be enough to rely solely on amplitude–asymmetric motion to generate lateral force and resist side gust perturbations. The negative value in Figure 13b indicates the direction of the averaged roll moment ${\bar{C}}_{M-\mathrm{roll}}$. According to the coordinate definition in Figure 1a, this negative moment will cause the body to roll to the side with a smaller flapping amplitude. ${\bar{C}}_{M-\mathrm{roll}}$ has the largest change rate when $\Delta \Phi^{*}$ is in the range of 0–0.2, followed by a slower increase as the speed increases further. When $\Delta \Phi^{*}$ is greater than 0.2, the absolute value of ${\bar{C}}_{M-\mathrm{roll}}$ still increases monotonously, but the slope decreases gradually. In the case of *f* = 5 Hz, ${\bar{C}}_{M-\mathrm{roll}}$ at $\Delta \Phi^{*}$ = 0.4 is 80.2% of ${\bar{C}}_{M-\mathrm{roll}}$ at $\Delta \Phi^{*}$ = 1. In Figure 13c, the average yaw moment ${\bar{C}}_{M-\mathrm{yaw}}$ changes rapidly when $\Delta \Phi^{*}$ is in the range of 0–0.4, followed by a slower increase as $\Delta \Phi^{*}$ increases further. ${\bar{C}}_{M-\mathrm{yaw}}$ reaches the maximum when $\Delta \Phi^{*}=1$. In the case of *f* = 5 Hz, ${\bar{C}}_{M-\mathrm{yaw}}$ at $\Delta \Phi^{*}$ = 0.4 is 64.2% of ${\bar{C}}_{M-\mathrm{yaw}}$ at $\Delta \Phi^{*}$ = 1.

So, these three aerodynamic components can be regarded as monotonically changing with $\Delta \Phi^{*}$. The minimum value is obtained when $\Delta \Phi^{*}=0$ and the maximum value is obtained when $\Delta \Phi^{*}=1$. Bilateral amplitude–asymmetric flapping motion will bring the maneuvering control forces of coupling roll moment and yaw moment. In order to perform more effective maneuvers based on amplitude asymmetry, it is recommended that $\Delta \Phi^{*}$ be kept in the range of 0 to 0.4, because adjustment within this range can cover most of the control torques that can be generated based on amplitude asymmetry. It can also obtain higher maneuverability by further increasing $\Delta \Phi^{*}$ and flapping frequency, *f*.

**Figure 14:**
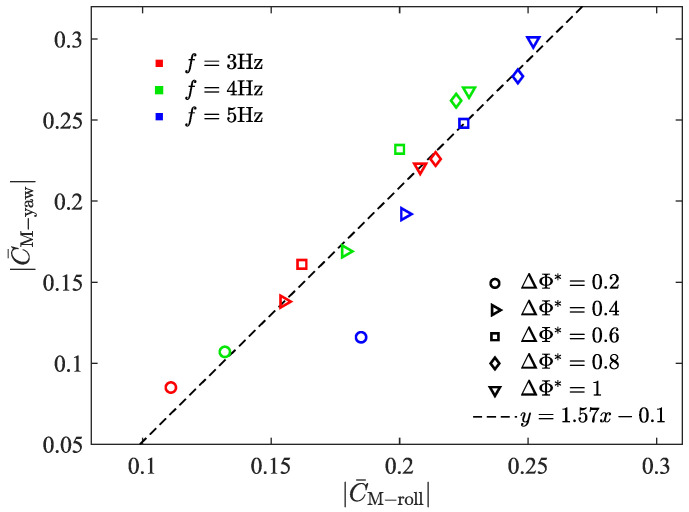
The relationship between $|{\bar{C}}_{M-\mathrm{roll}}|$ and $|{\bar{C}}_{M-\mathrm{yaw}}|$ generated by bilateral amplitude–asymmetric flapping motion.

In order to further explore the relationship between roll moment and yaw moment generated by this asymmetric flapping motion, $|{\bar{C}}_{M-\mathrm{roll}}|$ and $|{\bar{C}}_{M-\mathrm{yaw}}|$ measured at different *f* and different $\Delta \Phi^{*}$ are plotted (Figure 14). According to the obtained data points, the fitting is carried out, and the relationship between $|{\bar{C}}_{M-\mathrm{roll}}|$ and $|{\bar{C}}_{M-\mathrm{yaw}}|$ is as follows:

(16) $$|{\bar{C}}_{M-\mathrm{yaw}}|=p_{1}\left|{\bar{C}}_{M-\mathrm{roll}}\right|+p_{2},$$

where $p_{1}=1.57$, $p_{2}=-0.1$, SSE = 0.007, and R-square = 0.89. Different from the previous literature [31] that believed that amplitude asymmetry will mainly bring a single component of the control moment, the experimental results show that the coupling of the roll moment and yaw moment will be produced, and there is a good linear relationship between the generated roll moment and yaw moment.

### 3.3. Passive Pitching Deformation

**Figure 15:**
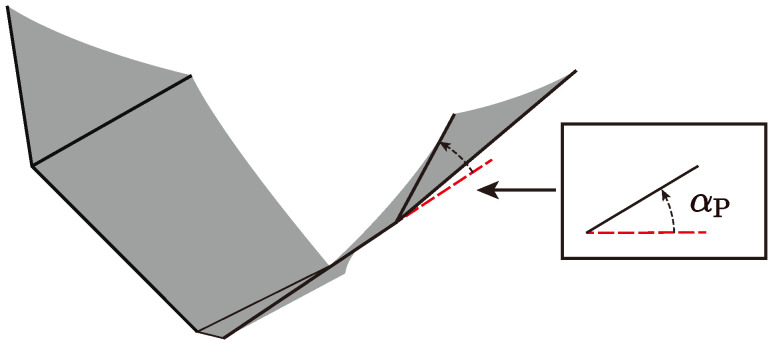
Definition of the passive pitching angle, $\alpha_{P}$; the red dashed line is the longitudinal direction. The angle is positive counterclockwise.

The passive deformation of the flexible wing is tracked using a high-speed video camera at a frame rate of 1000 fps. Because of the low aspect ratio of the flexible wing used in this study, twisting deformation is more significant than bending deformation. So, $\alpha_{P}$ is selected to measure the passive pitching deformation caused by the flapping motion. We define $\alpha_{P}$ as the passive pitching angle between Digit V of the wing and body’s longitudinal direction, as shown in Figure 15. The time history of $\alpha_{P}$ under different flapping amplitude, Φ, for flapping frequency, *f* = 5 Hz are measured. The data are smoothed by a Gaussian-weighted moving average filter.

**Figure 16:**
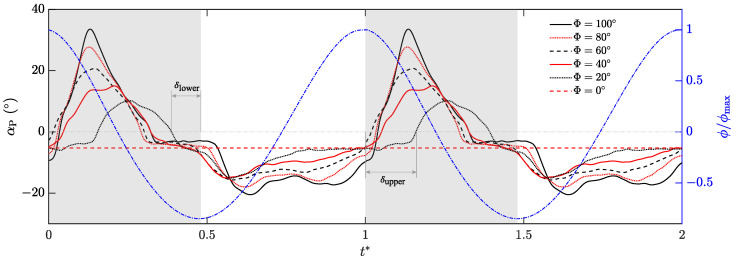
History of $\alpha_{P}$ for *f* = 5 Hz under different flapping amplitudes, Φ.

As shown in Figure 16, the red horizontal dashed line of Φ = 0° represents the constant angle value of the wing when it is not flapping and naturally drooping due to gravity. The maximum passive pitching angles for different $\Delta \Phi^{*}$ are reached around mid-downstroke. With the increase in Φ, the average wing tip velocity, ${\bar{U}}_{\mathrm{tip}}$, becomes faster, the peak point of $\alpha_{P}$ moves forward, and the duration of the peak decreases. In a single flapping-wing cycle, when Φ = 20°, $\alpha_{P}$ reaches the peak at $t^{*}$ = 0.26; when the flapping amplitude is 100, $\alpha_{P}$ reaches the peak at $t^{*}$ = 0.14. In late downstroke, $\alpha_{P}$ enters a flat region that is slightly larger than $\alpha_{P}$ for Φ = 0°. During the upstroke, the valley points move backward as Φ increases, and there will be two troughs of different magnitude. The first trough appears at the beginning of the upstroke, and the second trough appears at the end of the upstroke. The amplitude of the trough is significantly smaller than the amplitude of the peak. The gravity effect and the difference between the upper and lower wing surfaces may be the reasons for the difference in deformation during upstroke and downstroke.

**Figure 17:**
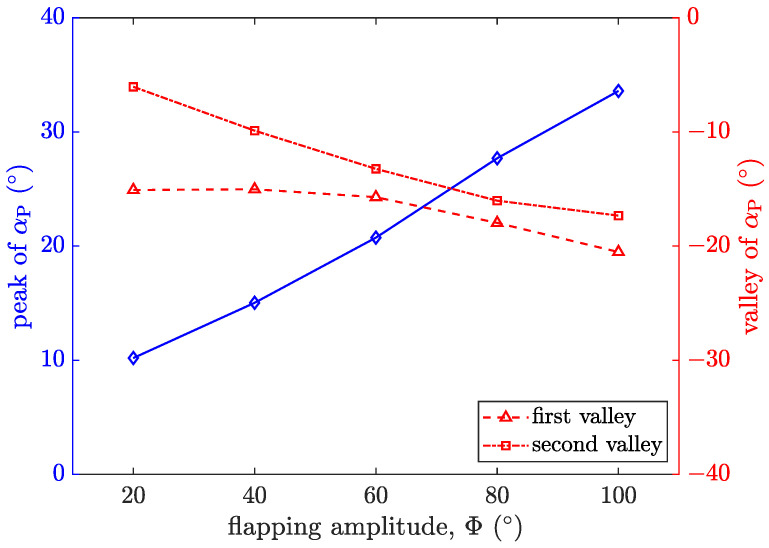
History of $\alpha_{P}$ for *f* = 5 Hz under different Φ. Blue represents the peak of $\alpha_{P}$, and red represents the valley of $\alpha_{P}$.

Figure 17 shows the variation in the peak and valley values of $\alpha_{P}$ with the flapping amplitude, Φ. It can be seen that the magnitude of the peak value increases nearly linearly with the increase in Φ. The growth rate of the peak value with Φ is significantly greater than the change rate of the valley value. The change rate of the second valley value with Φ is greater than that of the first valley value. As Φ increases, the gap between the two gradually decreases.

Figure 16 shows the changes in the flapping angle, ϕ, based on the active flapping motion, and the pitch angle, $\alpha_{P}$, based on the passive pitching motion in two cycles. It can be seen from the figure that there is a phase difference between flapping motion and pitching motion under different flapping amplitudes. Previous studies on insect flights [32,33] can be divided into three modes, according to the phase difference between translation and rotation: advanced, symmetric, and delayed modes. In this study, we define the phase difference δ between flapping and pitching motion as follows:

(17) $$\delta =\frac{t_{\alpha_{P}=0}-t_{\dot{\phi }=0}}{T},$$

where $t_{\alpha_{P}=0}$ is the time when $\alpha_{P}$ = 0, and $t_{\dot{\phi }=0}$ is the time of stroke reversal.

**Figure 18:**
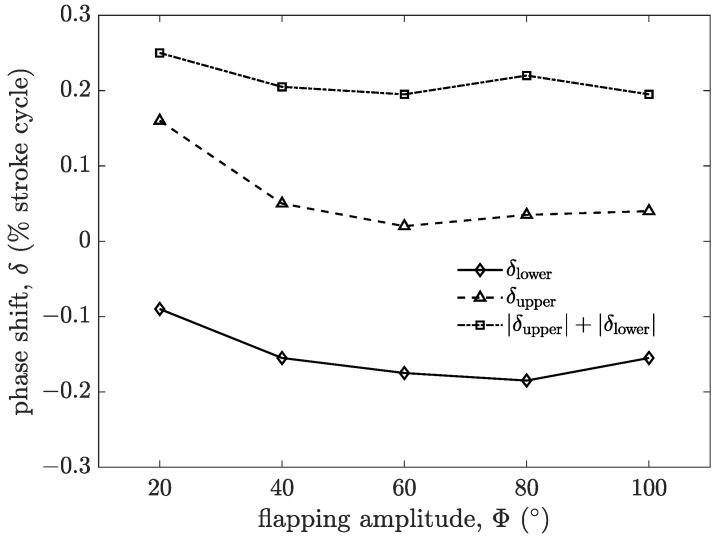
The relationship between the phase shift, δ, and flapping amplitude, Φ.

The relationship between the phase shift, δ, and flapping amplitude, Φ, is shown in Figure 18. $\delta_{\mathrm{upper}}$ is the phase shift at the upper reversal point, and $\delta_{\mathrm{lower}}$ is the phase shift at the lower reversal point, which are identified in Figure 16 for Φ = 20°. $|\delta_{\mathrm{upper}}\left|\;+\;\right|\delta_{\mathrm{lower}}|$ can be used to weigh the proportion of pronation and supination in the pitching motion cycle. In the measured flapping amplitude range, $\delta_{\mathrm{upper}}$ > 0, showing a delayed mode at the upper reversal point; $\delta_{\mathrm{lower}}$ < 0, showing an advanced mode at the lower reversal point. With the increase in Φ, $\delta_{\mathrm{upper}}$ decreases, which means the delayed mode begins to approach the symmetric mode at the upper reversal point. $|\delta_{\mathrm{lower}}|$ continues to increase with the increase in Φ, which means the advance is further intensified. This is mainly because the stroke plane of this experiment is set to be vertical, and the flexible wing is affected by the combination of inertia and gravity during the movement. Therefore, $|\delta_{\mathrm{upper}}\left|\;+\;\right|\delta_{\mathrm{lower}}|$ is only slightly fluctuating, and the change is not significant. It indicates that $|\delta_{\mathrm{upper}}\left|\;+\;\right|\delta_{\mathrm{lower}}|$ is not sensitive to the variation in Φ, and the proportion of pronation and supination in passive pitching motion cannot be adjusted by changing Φ.

## 4. Conclusions

In this paper, the flight features of natural creatures are extracted, and the aerodynamic characteristics of flapping wing motion with asymmetric amplitude are systematically studied. An experimental model with a pair of flexible membrane wings and two four-bar crank–rocker mechanisms is designed and constructed. Considering the actual situations of engineering applications, the bat-inspired flexible wing is simplified and lightweight. A dimensionless number, $\Delta \Phi^{*}$, used to measure the degree of amplitude asymmetry between bilateral wings is proposed. Then the aerodynamics and deformation are measured by force transducer and high-speed photography, respectively.

The experimental results indicate that transient aerodynamic forces are strongly dependent on $\Delta \Phi^{*}$ and *f*. With the increase in $\Delta \Phi^{*}$ or *f*, the transient force amplitude increases. And the time point of peak value moves forward as $\Delta \Phi^{*}$ increases, which is opposite to the change caused by the increase in *f*. In terms of cycle-averaged force, the bilateral amplitude–asymmetric flapping motion will mainly generate maneuvering control forces of coupling roll moment and yaw moment. $\Delta \Phi^{*}$ is recommended to remain in the range of 0–0.4 to perform more efficient maneuvers based on amplitude asymmetry. High-speed photography shows that the passive pitching deformation during the downstroke is significantly larger than that during the upstroke. The maximum passive pitching angle, $\alpha_{P}$, is reached around mid-downstroke and moves forward with the increase in Φ during a flapping cycle. There are two troughs of $\alpha_{P}$ during the upstroke. And the gap between the two gradually decreases as Φ increases. The proportion of pronation and supination in passive pitching motion cannot be adjusted by changing Φ.

This study produced data that are difficult to obtain from a live animal and has developed our understanding of the characteristics of kinematic asymmetries, aerodynamic forces, and passive wing deformation. These experimental results function as proof that asymmetric flapping motion can be implemented to create controllable aerodynamic forces, which have the potential to enhance the maneuvering performance of FWMAVs and develop control laws for maneuver flights. They can also be used to introduce or correct asymmetry in the flapping amplitude of contralateral wings when FWMAVs experience external perturbations or manufacturing and assembly errors, which may lead to inherent asymmetry of the aircraft.

## Figures and Tables

**Table 1:** Dimensions of wing planform geometry.

Variable	Value
Single wing length, *R* (mm)	186.6
Wingspan, *b* (mm)	385.3
Wing area, *S* (${\mathrm{mm}}^{2}$)	28,560
Mean chord, $\bar{c}$ (mm)	74.1
Aspect ratio	5.2

**Table 2:** Parameters of the flapping mechanism.

Structural Parameter	Length (mm)
$l_{0}$	59.9
$l_{1}$	64
$l_{2}$	15.5
$l_{3}$	5
$l_{4}$	16.2

**Table 3:** Length of the right crank, amplitude, maximum, minimum, and average flapping angle of the right wing under different $\Delta \Phi^{*}$.

$\Delta \Phi^{*}$	$r_{R}\left(\mathrm{mm}\right)$	$\Phi_{R}{(}^{\circ })$	$\phi_{R\max}{(}^{\circ })$	$\phi_{R\min}{(}^{\circ })$	${\bar{\phi }}_{R}{(}^{\circ })$
0	11.0	100	57.8	−41.7	8.0
0.2	9.4	80	45.0	−35.4	4.8
0.4	7.3	60	32.2	−27.4	2.4
0.6	5.0	40	20.9	−18.9	1.0
0.8	2.6	20	10.4	−10.0	0.2
1	0	0	−0.1	−0.1	−0.1

## Data Availability

The data supporting the findings of this study are available from the authors upon reasonable request.
